# A Comprehensive Survey of Recent Routing Protocols for Underwater Acoustic Sensor Networks

**DOI:** 10.3390/s19194256

**Published:** 2019-09-30

**Authors:** Tariq Islam, Yong Kyu Lee

**Affiliations:** Department of Computer Science and Engineering, Dongguk University-Seoul, Seoul 04620, Korea; tariqislam20@yahoo.com

**Keywords:** underwater sensor networks, routing protocols, survey, void avoidance, mobility

## Abstract

Underwater Sensor Networks (UWSN) have attracted huge attention due to their significance in oceanic observation and exploration. They offer a vast number of applications, many of which require routing the sensed data to a centralized location. This makes routing an important part of the design of such applications. In this paper, we present a comprehensive survey of recently proposed routing protocols for UWSNs. We evaluate the proposed schemes through an extensive set of parameters that define the core characteristics of a routing protocol. Moreover, we present a summary of the methods used by each scheme to familiarize readers with the basic operations of the schemes. We also present our view of the strengths and weakness of each scheme. For ease of description, the addressed routing protocols are divided into two categories: localization-based, and localization-free routing schemes. Each of the two categories is further divided into the protocols that consider node mobility, and those that do not. Lastly, we present our view on open research topics.

## 1. Introduction

Unlike terrestrial sensor networks, underwater sensor networks are faced with some unique challenges, such as the absence of GPS, three-dimensional node deployment, and mobility of nodes with water currents [1,2]. In addition to these challenges, the infeasibility of using radio waves as an underwater communication medium poses further challenges. Radio waves can propagate only short distances in water, as they fade quickly inside water, due to absorption. Long distance communication using radio waves requires very large antennae, which becomes a limiting factor in node design. Therefore, acoustic waves, which enable long distance communication inside water, are used as a medium of communication. However, the underwater acoustic channel also poses some challenges, such as higher levels of noise, Doppler effect, higher propagation delays, and higher bit error rates [3,4]. In order to be effective, protocol designs for underwater sensor networks must consider these characteristics of the underwater acoustic channel.

**Figure 1:**
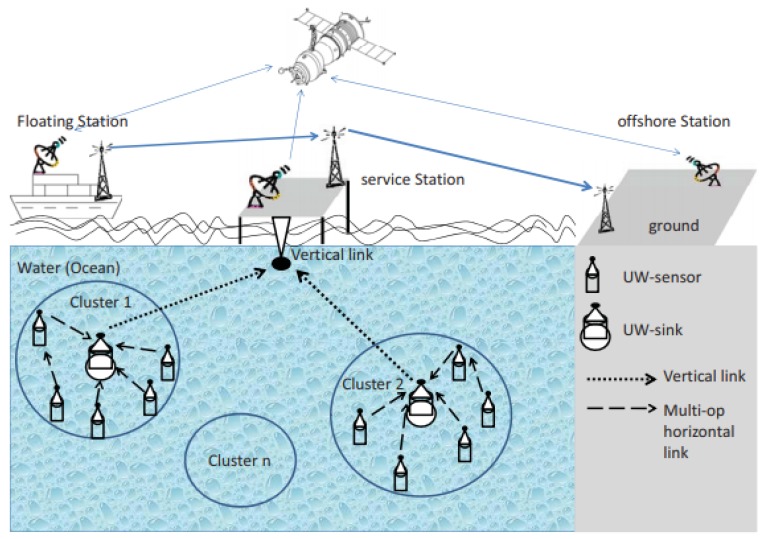
Underwater Sensor Network Architecture [10].

Figure 1 shows a generalized architecture of underwater sensor networks [5]. Sensor nodes, equipped with acoustic transceivers, are deployed underwater. These nodes can be static or mobile. The sensor nodes route data to the surface sink nodes, mostly using sensors at lower depth levels as relays. Alternatively, autonomous underwater vehicles (AUVs) can be employed to collect data directly from sensors. AUV-based routing requires efficient path planning for AUV travel. Moreover, employing AUVs incurs extra cost. 

Routing protocols hold a key position in the design of UWSNs. These protocols are responsible for determining efficient paths and route data along those paths from the sensor nodes to the stations where the data can be meaningfully interpreted. In order to achieve optimal performance, these protocols must be robust against the harsh underwater channel conditions. For example these protocols should efficiently deal with energy constraints, node mobility, high SINR, high end to end delays etc. 

Many surveys have been conducted on routing in underwater sensor networks [6,7,8,9]. However, most of these surveys do not focus on the recent work. Moreover, some of them do not discuss routing strategies and the merits and demerits of the protocols being surveyed. An account of these parameters is vital for the readers to attain a basic understanding of the protocols being reviewed. 

In this work, we present a review of recently proposed routing algorithms for underwater sensor networks. We analyze the protocols extensively, and identify their merits and demerits. In addition, our analysis includes an extensive set of evaluation parameters that have a direct impact on protocol performance. Moreover, we briefly explain the routing strategy of every protocol to familiarize the reader with the basic design of the protocol. The protocols are categorized based on location requirement: i.e., whether the protocol needs location estimates to carry out its operations or not. The protocols in each category are further divided based on mobility consideration i.e., whether a protocol considers node mobility or not.

In the following text, we briefly explain the issues a routing protocol design should address: 

*Energy Constraints:* Energy is a constraint in UWSN, as batteries can neither be recharged nor replaced easily. Therefore protocol designs must consider energy conservation.

*Connectivity Void Avoidance*: Connectivity voids are created if a node, which lies on a packet’s route from source to destination, goes down, due to energy drainage or some malfunction. A good routing protocol should avoid the creation of voids by load balancing. Moreover if voids are created, it should have strategies to work around the void regions, to successfully deliver packets to the destination node. 

*Location Estimation*: Due to the unavailability of GPS underwater, location estimation becomes a challenging and communication intensive task. A routing protocol that uses locations to set up paths must deal with extra communication overhead incurred due to localization.

*Mobility*: Underwater sensor nodes move with water currents. This movement may disrupt end-to-end paths and create connectivity voids if a node drifts out of the range of its adjacent sensor node(s) along the path from source to sink. Therefore while designing a routing scheme, free movement of nodes should also be considered.

*3D Deployment*: 3D deployment imposes further challenges, due to the addition of the third dimension, i.e., depth. Sensor nodes should be capable of relaying data to the sink nodes, forwarding it over multiple-hop paths. The nodes should adjust their depths in order to find a relay if there is no accessible relay using the current transmission range. However depth adjustment should ideally not create connectivity holes for other nodes.

In the following text we briefly explain all the parameters based on which we evaluated the routing protocols in this survey. Table 1 and Table 2 list all of these parameters, and their values for each of the considered protocols:

*Mobility*: This parameter includes both limited and free mobility consideration.

*Location Requirement*: Indicates the requirement of knowing the geographical locations of sensor nodes for successful operation of the scheme.

*Main consideration*: Indicates the main issue addressed by the proposed scheme.

*Next Hop Selection Criteria*: Indicates the criteria for selecting forwarding nodes. The next hop selection criteria determines the capability of the proposed scheme for addressing different issues related to routing. For example if “Residual energy” is considered, the aim is balanced energy dissipation of the node. Considering “Link condition or SNR” aims at improving reliability, etc.

*Clustering*: Indicates whether the proposed scheme is a cluster-based scheme or not. Clustering offers advantages, such as low communication overhead, small contention domain, and transmission with low transmission power, etc. 

*Connectivity Void Handling*: Connectivity void handling includes two issues: (1) Prevention of void creation, e.g., by balancing energy dissipation; (2) Ability to work around voids to successfully deliver packets, for example, by using detouring techniques.

*Sink*: Indicates whether the scheme uses a single-sink or multi-sink architecture. Both architectures have their own advantages and disadvantages. For example in the case of multi-sink architecture, the receiving plane is widespread. This helps in load distribution, and provides flexibility in path selection. Whereas in the case of single-sink architecture, all the traffic is directed towards the same destination, thus burdening certain nodes near the sink and shrinking path options. However, single-sink architecture offers less complexity, compared to multi-sink architecture. 

*Deployment*: Indicates whether authors have considered 2D or 3D deployment.

The rest of this paper has been organized as follows: Section 2 presents a detailed survey of the routing protocols considered. It is further divided into two sub-sections dividing the routing algorithms into location-based routing algorithms, and location-free routing algorithms. Section 3 presents the conclusion, while Section 4 discusses open issues and challenges.

## 2. Characteristics of the Underwater Acoustic Channel

### 2.1. The Speed of Sound in Water

The speed of sound in oceans depends on three main factors namely; temperature, salinity and depth. Equation (1) presents the empirical formula given by Mackenzie to estimate the speed of sound in water.

(1) $$\begin{array}{ll} c= & 1449+4.591T-5.304\times 10^{-2}T^{2}+2.374\times 10^{-4}T^{3}+1.34\left(S-35\right)\\ & +1.63\times 10^{-2}D+1.675\times 10^{-7}D^{2}\\ & +1.025\times 10^{-2}T\left(S-35\right)-7.139\times 10^{-3}TD^{3} \end{array}$$

### 2.2. Attenuation

Pathloss of acoustic signals in the underwater acoustic channel is given by Equation (2). The path loss is a function of frequency *f* and distance *d* in kilometers:

(2) $$A\left(d,f\right)=d^{S}.\alpha {\left(f\right)}^{d}$$

where *S*, d, $d_{ref}$ and α(f) are the spreading factor, distance and absorption coefficient, respectively. The path loss is given in dB using Equation (3):

(3) 10logA(d,f)=S.10 log d+d.10 log α(f)

The geometry of a transmitted acoustic signal is described by its spreading factor (*S*). Typical values of *S* are 2 for spherical spreading, 1 for cylindrical spreading and 1.5 for practical spreading.

The absorption coefficient or α(f) can be computed using the formula give in Equation (4) which expresses absorption coefficient in dB/km for *f* in kilohertz as [4]:

(4) $$10\log\;\alpha \left(f\right)=0.11\frac{f^{2}}{1+f^{2}}+44\frac{f^{2}}{4100+f^{2}}+2.75.10^{-4}f^{2}+0.003$$

The formula in Equation (4) is generally effective when the frequency *f* is higher than a few hundred Hz. Equation (5) can be used for lower frequencies:

(5) $$10\log\;\alpha \left(f\right)=0.002+0.11\frac{f^{2}}{1+f^{2}}+0.011f^{2}$$

**Figure 2:**
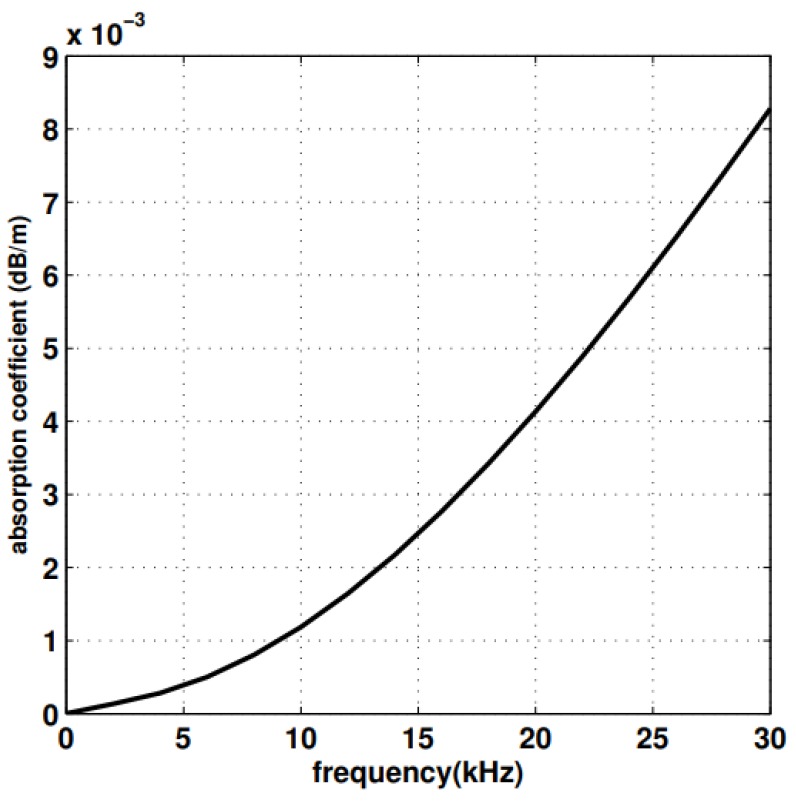
Absorption coefficient as a function of frequency [11].

Figure 2 [11] shows absorption coefficient as a function of frequency. α(f) increases rapidly with the increase in frequency, thus resulting in a bound on the maximum usable frequency for a link between nodes with a given distance *d* between them. 

### 2.3. Noise

The ambient noise in an acoustic channel is modeled using four constituent sources: shipping activity noise (Equation (6)), turbulence (Equation (7)), breaking waves (Equation (8)), and thermal noise (Equation 9). Equations (6)–(9) give the power spectral density of the four noise sources in dB re μ Pa per Hertz for frequency *f* in kilohertz:

(6) $$10\log N_{s}\left(f\right)=40+20\left(s-0.5\right)+26\log f-60\log\left(f+0.03\right)$$

(7) $$10\log N_{t}\left(f\right)=17-30\log f$$

(8) $$10\log N_{w}\left(f\right)=50+7.5w^{1/2}+20logf-40\log\left(f+0.4\right)$$

(9) $$10\log N_{th}\left(f\right)=-15+20\log f$$

The power spectral density of the overall ambient noise in underwater acoustic channel is given by Equation (10):

(10) $$N\left(f\right)=N_{s}\left(f\right)+N_{t}\left(f\right)+N_{w}\left(f\right)+N_{th}\left(f\right)$$

The signal to noise ratio (in dB) experienced at a receiver in the underwater acoustic channel is given by Equation (11):

(11) SNR=SL−TL−NL−DI

where, *NL* represents the level of ambient noise in dB, *TL* represents the transmission loss (dB), *DI* or directivity index is set to 0. *SL* or source level of the transmitter in dB can be computed using Equation (12) as follows:

(12) $$SL=10\log\left(\frac{I_{t}}{0.67\times 10^{\left(-18\right)}}\right)$$

where *I_t_* represents the intensity of transmission power. For shallow waters, *I_t_* can be calculated in Watts/m^2^ using Equation (13):

(13) $$I_{t}=\frac{P_{t}}{2\times \pi \times 1m\times z}$$

In case of deep waters, Equation (14) can be used to calculate *I_t_*:

(14) $$I_{t}=\frac{P_{t}}{4\times \pi \times 1m\times z}$$

## 3. Routing Protocols for UWSNs

**Figure 3:**
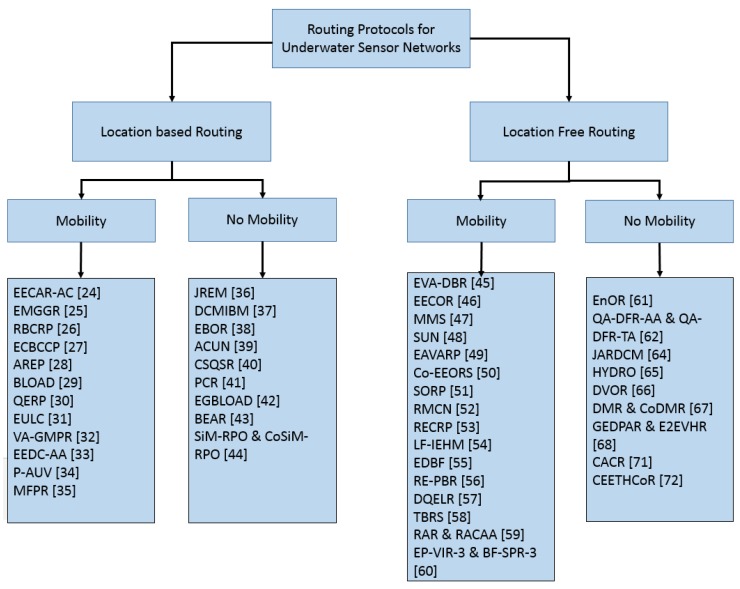
Classification of routing protocols based on location requirement and mobility.

This section presents detailed analysis of all the routing schemes considered in this survey. The protocols have been divided into two categories based on their localization requirement, i.e., localization-based routing schemes, and localization-free routing schemes. Each of the two categories are further subdivided into protocols that consider mobility (limited or free), and those that do not consider mobility of sensor nodes (Figure 3).

### 3.1. Location-Based Routing Schemes 

Location-based routing schemes require location estimates of sensor nodes to determine routes for data forwarding. Location estimation can be achieved using suitable localization services, such as those proposed in [12,13,14,15,16,17,18,19,20,21,22,23]. Table 1 analyzes these protocols based on a number of parameters explained in Section 1. Location-based routing schemes can be categorized based on whether or not they consider node mobility.

#### 3.1.1. Location-Based Routing with Mobility Consideration

This section surveys the location-based routing schemes that consider limited or free mobility of sensor nodes, relay nodes, and/or sink nodes. 

##### EECAR-AC

**Figure 4:**
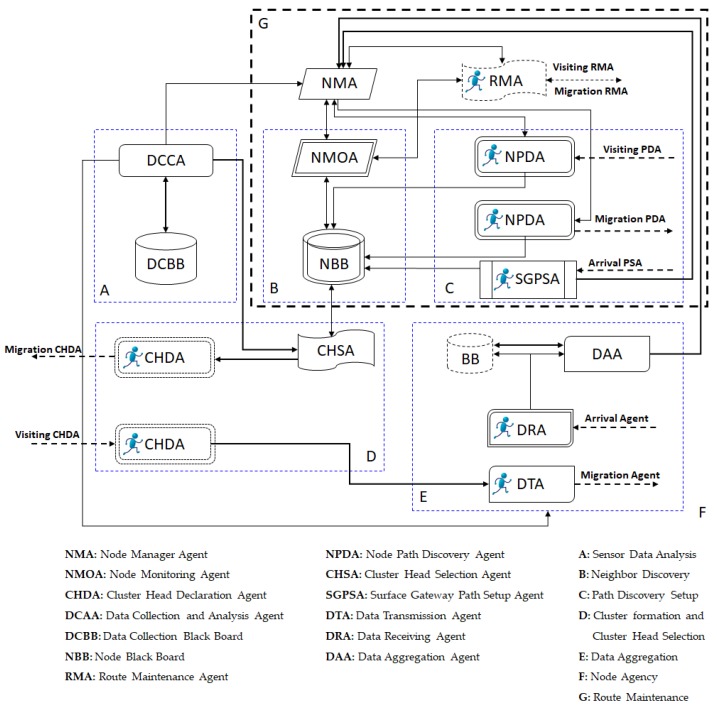
Node agency.

EECAR-AC [24] is an event-driven cluster-based approach for routing in UWSNs. In this scheme, routing is carried out with the help of agents implemented in sensor nodes, AUVs, surface gateways, and underwater gateways (Figure 4). The scheme has two phases: (1) route discovery phase, and (2) route maintenance phase. Route discovery deals with the selection of relay nodes from the source to a surface gateway. The selection of relay nodes is based on link quality, depth, residual energy levels, hop count, and queue size. Data transmission is initiated as soon as the paths are set up. The route maintenance phase starts after paths are set up, and data transmission has started. This phase ensures the availability of alternate paths from the source to surface gateways in the case of link breakages or node malfunction. If the network gets partitioned i.e., alternate paths through regular relay nodes are not available, AUVs are employed to act as relays. Route maintenance improves the chances of successful delivery; however, it involves multiple transmissions of control packets, which increases communication overhead. The proposed scheme achieves reliability, load balancing, and end-to-end delay by the inclusion of link quality, residual energy, and hop count, respectively, as the next hop selection criteria. 

##### EMGGR

EMGGR [25] has three main components: (1) gateway election procedure, (2) gateway information update procedure, and (3) data packet forwarding procedure. The gateway election procedure deals with the selection of a gateway or relay node for each cell of the logical grid (the network area is partitioned into a three-dimensional logical grid). Gateway selection is based on a value “W” that is based on the remaining energy level of the candidate node, and its distance from the center of the cell. The node that maximizes W is selected as gateway for its cell. Selection of a node as gateway based on its distance from the center of the cluster and residual energy ensures avoidance of burdening one node with the role of gateway node, thus achieving balanced energy dissipation, and reducing the chances of void creation. After selection, every gateway periodically checks its remaining energy level. If its remaining energy level is higher than a certain threshold value, it transmits an update message, and continues to work as gateway (the update message resets the “existence timer” (maintained by ordinary nodes) for this gateway). However, if its residual energy level is lower than the threshold value, the gateway silently abandons its role as gateway. A new election process is carried out upon expiry of the existence timer. The third component of EMGGR is a data packet forwarding mechanism that can be divided into two parts: (1) construction of node disjoint paths, and (2) Packet transmission through node disjoint paths via gateways. Construction of node-disjoint paths increases the reachability of the network. Moreover, parallel packet transmission over multiple node-disjoint paths adds reliability and immunity against connectivity holes. The downside of this protocol is the need for geographic coordinates that adds extra overhead. Moreover, it has high end-to-end delay, due to the higher number of hops between source and sink. 

**Table 1:** Survey of localization-based routing protocols.

Reference	Main Consideration	Next Hop Selection Criteria	Mobility	Location Required	Clustering	Connectivity Void Handling	Sink	Deployment
EECAR-AC [24]	Void Avoidance, Network Lifetime, E2E delay.	Node energy, propagation delay, hop count, channel quality	Yes	Yes	Yes	Yes	Multiple Sink	3D
EMGGR [25]	Reliability, Void Avoidance.	Pre-determined multiple node disjoint paths	Yes	Yes	No	Yes	Single-sink (Multi-sink can also be used)	3D
RBCRP [26]	Load Balancing, reduce outage probability of relay nodes.	SNR, depth, residual energy	Yes	Yes	No	No	Multiple Sinks	3D
ECBCCP [27]	Energy conservation, Reliability.	Link quality and hop count	Yes	Yes	Yes	No	Multiple Sinks	3D
AREP [28]	Void handling, Link asymmetry.	Symmetry of link, Distance from the destination	Yes	yes	No	Yes	Single-sink	3D
BLOAD [29]	Energy Holes avoidance, balanced energy consumption.	Distance from sink	Yes	Yes	No	No	Single-sink	2D
QERP [30]	Achieve high packet delivery ratio (PDR), low end-to-end delay, and improve network wide energy consumption for real time applications.	Link quality, shortest path	Yes	Yes	Yes	Yes	Single-sink	3D
EULC [31]	Hot Spot mitigation, Balanced Energy dissipation, Improved network lifetime.	Residual Energy, Distance from the candidate forwarder to the sink node, distance from the current sender to the candidate forwarder	Yes	Yes	Yes	Yes	Single-sink	3D
VA-GMPR [32]	Reliability, Load balancing, void avoidance.	Optimality of Path length	Yes	Yes	No	Yes	Single-sink	3D
EEDC-AA [33]	Balance energy consumption and prolong underwater network lifetime. Prioritize collected data based on its importance.	Available Energy	Yes	Yes	No	No	Multi-sink	3D
P-AUV [34]	Energy efficiency, Low latency.	Distance to the sink node	Yes	Yes	No	No	Multi-sink	2D
MFPR [35]	Identify optimal energy-efficient routing coverage set.	Optimal route selection based on location of nodes and available energy	Yes	Yes	No	No	Multi-sink	3D
JREM [36]	Increase network lifetime by avoiding Energy Holes and balancing energy consumption.	Probability of Successful reception, Load Weights (derived to achieve balanced energy consumption)	No	Yes	No	Yes	Single-sink	2D
DCMIBM [37]	Propose an optimal node placement scheme and a clustering scheme to increase lifetime of the network by controlling energy consumption.	CHs act as relays. CHs are selected based on available energy and location of candidate sensor node within its cluster	No	Yes	Yes	NA	Single-sink	3D
EBOR [38]	Energy consumption, network lifetime Reliability, PDR.	Residual Energy, Packet delivery probability Efficient transmission distance	No	Yes	No	No	Multi-sink	3D
ACUN [39]	Selection of appropriate node as CH based on residual energy distance from sink, Selection of appropriate next hop to minimize energy consumption.	Estimated Energy Consumption of the sender (based on distance from the candidate node)	No	Yes	yes	yes	Single-sink	3D
CSQSR [40]	Guarantee application-specific QoS, while also maximizing the network lifetime.	Node Position/Location Throughput (QoS parameters)	No	Yes	No	No	NA	3D
PCR [41]	Reliable and energy-efficient data delivery using combination of Transmission Power control and opportunistic routing.	Reduction in overall energy cost, Improvement in Packet Delivery Probability	No	Yes	No	Yes	Multi-sink	3D
EGBLOAD [42]	Load balancing, void management.	Available energy and distance of the forwarder from sink	No	Yes	No	Yes	Multi-sink	3D
BEAR [43]	Mitigating imbalanced and inefficient energy consumption.	Residual energy, depth	No	Yes	No	Yes	Single-sink	2D
RPO [44]	Energy Efficiency, Reliability.	NA	No	Yes	No	NA	Multi-sink	2D

##### RBCRP

**Figure 5:**
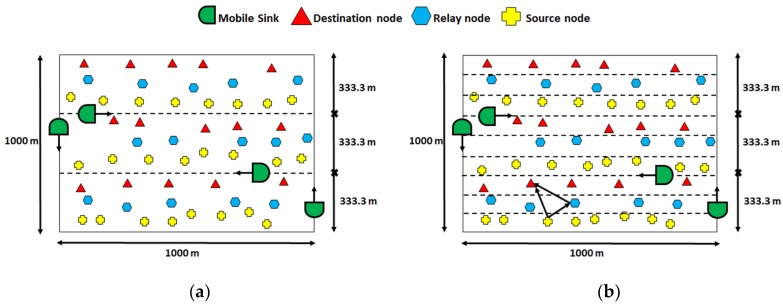
Division of a network area into regions and sub-regions. (**a**) Division of field into regions; (**b**) Division of field into sub-regions.

RBCRP [26] is a cooperative routing scheme that works in two phases. During the initial setup phase, nodes discover neighbors (potential forwarders) by broadcasting “hello” packets. The hello packets contain the SNR value, energy level, depth, and ID of the sender. Relay nodes are selected based on these parameters with the help of a function that aims to diminish outage likelihood of relays by balancing data load. The setup phase is followed by a steady state phase, during which packets are forwarded to selected relay nodes. Retransmission is requested if BER (calculated at the destination) is higher than a predefined threshold value. In order to improve packet delivery ratios (PDR), RBCRP employs mobile sink nodes. These mobile sink nodes traverse the entire network by following horizontal and vertical traversal paths (Figure 5), thus minimizing communication distance for transmitting nodes, thereby reducing packet drop rates, and improving total throughput and network lifetime. The performance of RBCRP with regard to network lifetime and throughput depends mainly on the SNR threshold value and number of relay/forwarder nodes. The proposed scheme achieves reliability, load balancing, and end-to-end delay by the inclusion of SNR, available energy, and depth, respectively, as the next hop selection criteria. The downside of this scheme is that in order to function properly, it requires the locations of the nodes. Location estimation, which is a communication intensive process, increases the overall communication overhead.

##### ECBCCP

**Figure 6:**
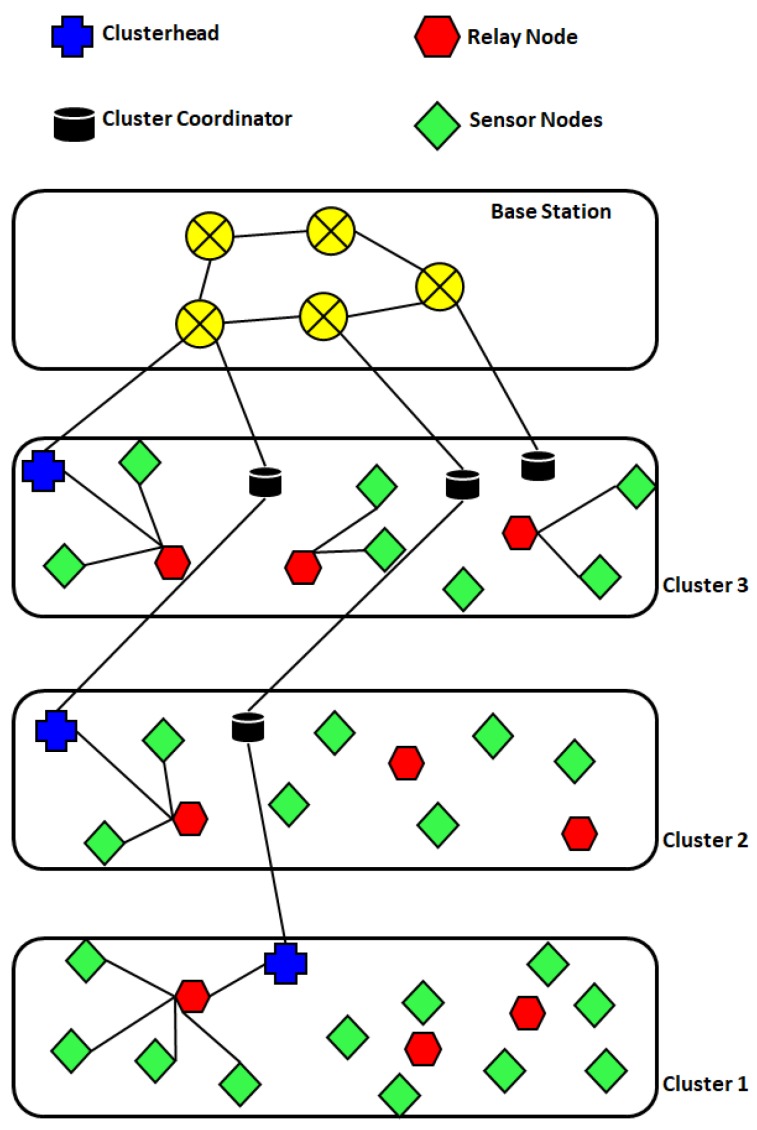
Network model.

ECBCCP [27] is a cluster-oriented routing scheme. This divides the network into sub-regions, each of which is assumed to be a cluster. Every cluster contains a CH, cluster coordinators, relay nodes, and ordinary sensor nodes (Figure 6). The cluster head collects data from nodes located in its cluster through relay nodes, and passes the data on to the cluster coordinators in the adjacent upper clusters. The same process is followed in each cluster, until the packet reaches the sink node(s). In ECBCCP, Cluster heads and relay nodes can store data. This helps in avoiding the retransmission of already received data. A newly received packet is compared with the already saved packets. In the case of a match, forwarding of the received packet is cancelled. Instead, a control message, (which is much smaller than the data packet), is sent to the sender to inform it about the current status. However, if the new data packet is an updated version of the previously stored data, the saved data is updated, and the newly arrived packet is forwarded. Relay nodes are selected based on a confidence value, which is a function of the link quality and hop count. In the case of a tie, i.e., two nodes having the same confidence value, the node with higher level of energy is selected as relay node. ECBCCP reduces unnecessary traffic by cancelling the transmissions of previously received data. However, the control messaging incurred for communication between different communicating entities and for informing the senders about status of their packets increases its communication overhead. 

##### AREP

**Figure 7:**
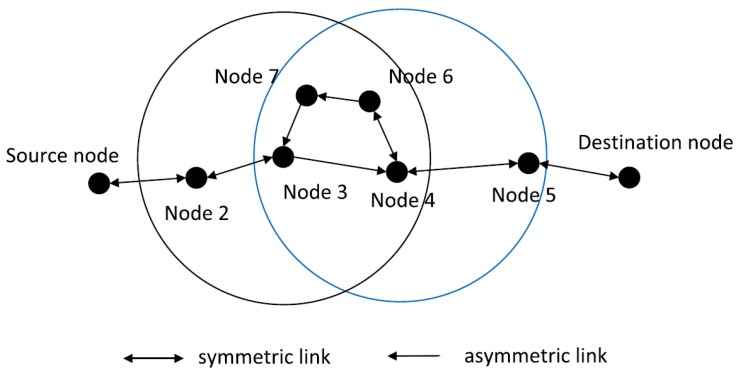
Greedy route establishment phase.

AREP [28] is designed to address the one-way communication problem that arises due to the asymmetry of links between senders and receivers in the underwater environment. The protocol achieves its task in four phases, termed: link state identification, greedy route establishment, void elimination, and routing update. Every node in the network builds up neighborhood tables during network initialization. The information in the tables is used during the link state identification stage to determine the symmetry between a node and its neighbors. This is followed by the greedy route establishment phase (Figure 7), during which the nodes that have bidirectional links with the sender are chosen as candidate relays. The node among the candidate forwarders that is nearest to the destination node is chosen as the forwarder. AREP eliminates the void problem with the help of notification messages that are sent from the void node to the upstream node. Route updates are made to deal with topology changes due to node mobility. If as a result of mobility, a void node is no longer a void node, the upstream nodes update their neighbor tables to include the previously void node. AREP improves delivery ratio by proposing a smart method for relay selection. Moreover, it adapts itself quickly to changes in network topology due to mobility. However, continuous table maintenance and updates incur relatively high communication overhead.

##### BLOAD

BLOAD [29] focuses on resolving the energy hole problem by balancing energy consumption among nodes located at different distances from the sink node. It has three phases. Phase one deals with deployment. Nodes are deployed in multiple circular regions (also called coronas) around a sink node. Each corona has equal width, and each node in a corona has equal distance from the sink node. After deployment, load weights corresponding to every corona are calculated in the 2nd phase. The load weights determine the transmission range allocation to nodes in different coronas to achieve balanced energy dissipation. In phase three, nodes transmit data using the assigned transmission range. As BLOAD solves the energy sink hole problem by allowing the nodes farther from sink to transmit with high transmission powers, it exposes deeper nodes to rapid energy drainage, as they transmit with high transmission power. Moreover high transmission power also increases interference and contention. 

##### QERP

QERP [30] is a cluster-based quality of service aware routing protocol. It aims to mitigate the effects of underwater channel impairments to provide QoS guarantees, such as high packet delivery ratio, low delays, etc. To achieve this end, QERP uses a small clustering technique, in which sensor nodes are connected into a hierarchy, which helps in even distribution of energy and traffic load. Routing path selection and forwarding decisions are made in a greedy manner, keeping in view the link quality between clusterheads. Moreover, the use of quality of service aware shortest path selection algorithm enables reduction in route loops, thereby reducing network delays, and achieving better energy efficiency. Furthermore, failure of a forwarding node is dealt with by adjusting transmission power based on information from routing tables, thus avoiding connectivity voids. QERP improves packet delivery ratio and reliability by selecting relays based on link condition and the shortest distance to sink node. However, due to the relatively higher load, clusterheads may consume their energy quickly, thus creating energy holes.

##### EULC

EULC [31] is a cluster-based routing scheme. In EULC, the network is divided into layers of unequal width, with the shallower layers having less width compared to deeper layers. This layering strategy mitigates the “hot spot” problem by regulating transmission power levels based on a node’s distance from the sink node. Clusters are formed within each layer. Clusterheads are selected depending upon the remaining energy of the candidate node, degree of the candidate node (which is a function of the neighboring nodes of the candidate node), and its distance from sink. Data transmission starts after setting up clusters and clusterheads. Routing is done taking into account the distance between the sender and a candidate forwarder, and the residual energy level of the candidate forwarder. EULC increases network life by setting contending radii of CHs based on its distance from sink, thus reducing cluster scale near the sink. Moreover it balances energy dissipation by selecting CHs based on residual energy level. 

##### VA-GMPR

VA-GMPR [32] works by dividing a UWSN into a virtual three-dimensional grid of equal sized cubic cells. Each cell has an elected gateway. Each gateway node hosts a gateway table that contains information about gateways up to two hops away. This information is used by the grid void avoidance scheme to avoid connectivity voids. Moreover, each sensor node hosts a path table that contains four cell disjoint paths from the underwater sensor node to the surface sink node. Traffic load balancing is achieved by the implementation of round robin selection of paths by each source gateway. In order to deal with connectivity holes or voids, a void avoidance method is proposed. The void avoidance technique used is based on the magnitude of the void area. The techniques used are hole bypassing, path diversion, and path back tracking, which solve lowest-to-highest complexity voids in the presented order. VA-GMPR enhances reliability by considering multiple node disjoint routes connecting a source node to the destination. Out of the available paths, longer and infeasible paths are dropped, thus saving network resource, and improving E2E delay. However, the selection of a few optimal paths out of many available paths introduces complexity.

##### EEDC-AA

EEDC-AA [33] is an energy efficient data collection protocol that assumes a network consisting of three kinds of nodes: (1) Sensor nodes that sense the phenomenon under consideration, (2) collector nodes that gather data from sensor nodes (through single or multihop communication), and AUVs, which gather data from collectors, and offload it upon surfacing. Moreover, the protocol uses two types of underwater communication media. Acoustic communication is used between sensor nodes and collectors, while optical communication is used between collectors and AUVs. The proposed scheme aims to minimize the energy efficiency and avoid connectivity holes, while performing data gathering based on a value of information metric. Value of information is strongly connected to time within which the data can be considered useful. Therefore, data with higher value of information is given preference in the data gathering process. Relays are selected depending upon the available energy. A node is chosen as relay if its available energy level is above a certain threshold level. EEDC-AA proposes a two-phase data gathering mechanism. The first phase deals with routing the collected data from sensor nodes to sink nodes. Meanwhile, the second phase is related to value of information- based path planning for a AUV to retrieve data from underwater sink nodes. EEDC-AA can prioritize data based on the value of information, and can respond to critical events on a priority basis. However, it does not explain how value of information, which is an important metric, is estimated. The proposed scheme improves network lifetime by reducing the chances of void creation through load balancing.

##### P-AUV

P-AUV [34] is a routing protocol designed for *ad hoc* AUV-based networks. This aims to achieve energy efficiency and higher packet delivery ratios by minimizing collisions. The proposed protocol uses locations of nodes for forwarding decisions, and creates loop-free paths in which nodes near the sink nodes have priority to be chosen as relays. Collisions are reduced by configuring every sensor node to select a back-off value, based on its location. All AUVs are equipped with the necessary apparatus for navigation. Thus information about initial position combined with navigation enables AUVs to know their current position at any point of time. Moreover, synchronization between AUVs is not required, hence reducing synchronization-related communication overhead. An AUV that is acting as a relay makes its forwarding decision based on its location. It selects a back-off value based on its distance from the source node i.e., the AUV with greater distance from a source node has priority to decrease its packet delivery delay, compared to the AUV with closer distance from its source node. Besides reducing collisions, communication overhead is further reduced by avoiding the exchange of state information, synchronization, neighborhood, and location details. By reducing communication overhead, P-AUV not only reduces energy consumption, but also achieves improved delivery ratio and small end-to-end (E2E) delays. P-AUV does not use GPS hardware. Moreover, sensor nodes are not required to surface, in order to refresh their coordinates. Initial position information combined with displacement information from tracking apparatus is used by AUVs to update their positions. However, this introduces position estimation error that may affect routing, as routing decisions are based on the self-location estimation of a node.

##### MFPR

**Figure 8:**
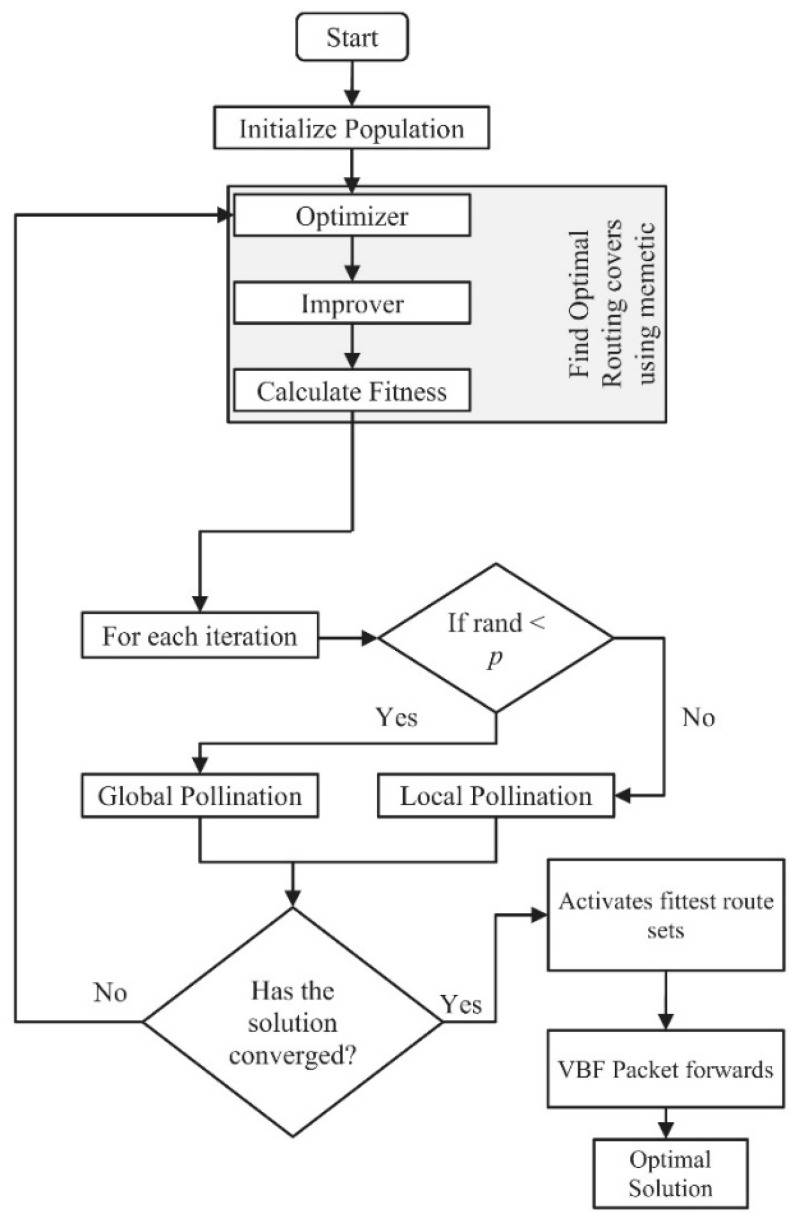
The proposed scheme [45].

MFPR [35] is a QoS-aware routing scheme that uses memetic flower pollination (MFP) to determine an optimal energy efficient forwarding set of underwater sensor nodes in a multiple sink architecture (Figure 8). The scheme performs its operations in two phases: (a) MFP identifies the set of nodes forming the optimal route from source node to the sink. The preliminary population uses optimal forwarding nodes to create a possible set of relays forming a route from the source node to the destination node. The MFP routes the populated paths according to best fitness value. (b) A flower pollination algorithm (FPA) switches between global and local pollination, in order to relay packets on reliable routes. MFPR employs grant free pattern division multiple access (GFPDMA), which reduces latency and transmission delay. MFPR delivers packets optimally with high energy efficiency and low end-to-end delay, while achieving longer network lifetime and high packet delivery ratio. Moreover MFPR deploys replaceable nodes near the sink to overcome the voids created due to the quicker dissipation of the top layer node. However this seems to move the same problem one hop down.

#### 3.1.2. Location-Based Routing without Mobility Consideration

This section surveys the location-based routing schemes proposed for underwater sensor networks with static node deployment.

##### JREM

JREM [36] is designed to avoid energy holes, and to achieve a fair balance of energy consumption among sensor nodes. It assumes manual deployment of nodes based on a pre-defined pattern that is aimed at balancing energy dissipation across the network. The authors prove that the transmission load can be evenly distributed if sensor nodes adjust their transmission powers keeping in view the deployment pattern. The transmission power can be adjusted by switching between n numbers of transmission power levels. The number of transmission power levels i.e., n, helps in determining the set of potential next hops and their load weights, thus resulting in fair energy dissipation in the network. Keeping in mind the importance of the number of transmission power levels (*n*) for fair energy consumption, the authors derive the optimal value of *n* that maximizes the life span of the network by overpowering the energy holes issue. JREM improves network lifetime by distributing the network load evenly, and by avoiding the energy hole problem. However, a drawback of this scheme is that it needs pre-defined deployment patterns, thus reducing its scope. Moreover, deployment cost in deep waters may increase, as all nodes are anchored.

##### DCMIBM 

DCMIBM [37] is a cluster-based data gathering scheme for magnetic induction based monitoring in underwater environments. Nodes are deployed in hexagonal fashion, and clusters are formed in which certain nodes are selected as cluster heads. Cluster head selection is based on the residual energy levels of the nodes, and their location within the cluster. The node with highest energy levels and best location, i.e., center of the cluster, is chosen as the cluster head. Data collection is carried out with the help of an AUV. Ant colony optimization is used to search for shortest routes for AUV traversal, thus reducing the end-to-end delays. The proposed scheme reduces end-to-end delays, and improves network lifetime through better energy management. 

##### EBOR

Reference [38] proposes a DS evidence theory-based opportunistic routing scheme (EBOR) to enable energy efficient forwarding with improved end-to-end delays and packet delivery ratios. The forwarding decisions are made based on tradeoff between three metrics: packet delivery probability, efficient transmission distance, and residual energy. To improve energy efficiency, the number of forwarders on each hop is reduced by optimizing the number of forwarders based on a trust-based computation. This also enables fair distribution of load between relay nodes, thus achieving uniformity in energy dissipation. EBOR reduces collisions (and therefore retransmissions) by setting different holding times for different relay nodes. This results in lower communication overhead, and better energy efficiency. Moreover, it also achieves fair energy dissipation by using residual energy as a criterion for forwarder selection, thus prolonging network lifetime. Moreover, reliability is achieved by including link quality in the forwarder selection criteria. Equation (15) presents the holding time of the *j*-th node in the forwarding set *i*:

(15) $$T_{f_{i,j}}=\frac{d\left(n_{i,}f_{i1,}\right)}{C}+\overset{j-1}{\underset{p=1}{\sum }}\frac{d\left(f_{ip,}f_{i,p+1,}\right)}{C}-\frac{d\left(n_{i,}f_{ij,}\right)}{C}+j\times T_{proc}$$

where *C* and $T_{proc}$ are speed of sound in water and packet processing time.

##### ACUN

**Figure 9:**
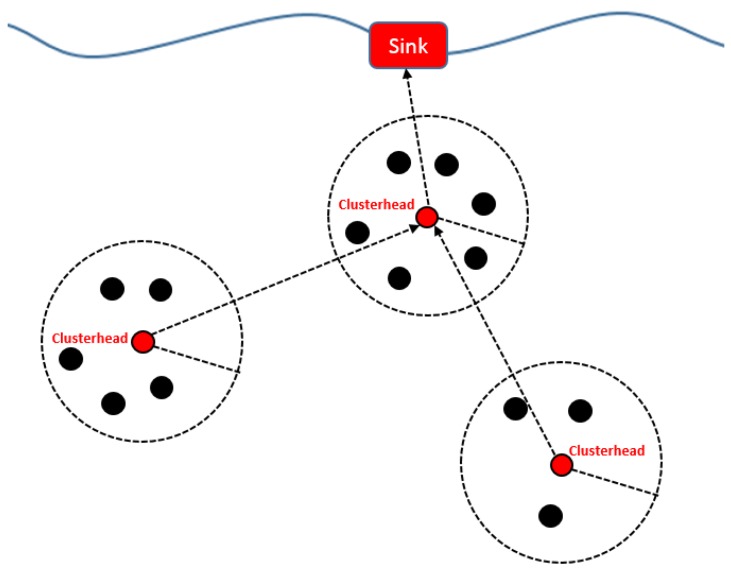
Network Model.

Reference [39] proposes ACUN, which is a cluster-based routing protocol. ACUN divides the nodes into a hierarchy, taking into account the distance between CHs and sink (Figure 9). It determines the size of the competition radius of CHs based on their residual energy levels. CHs are selected based on their residual energy levels. A node that has high level of residual energy becomes a candidate clusterhead. ACUN avoids early demise of a CH with bigger competition radius, i.e., the proposed protocol can avoid the situation when due to greater distance between a CH head and sink node, the CH transmits with high transmission power, resulting in the CH dying earlier than the rest of the network, thus creating voids in the network. Routing is performed in a multihop fashion, instead of a single hop transmission. This multi hop routing favors nodes that are located farther from the sink, as they can transmit with smaller transmission power compared to the one hop transmission case, in which they would have required much higher transmission power, thus consuming more energy. ACUN improves the overall network level energy consumption and network lifetime. ACUN avoids the premature death of CH near the sink, by adjusting CH competition radius adaptively based on distance from the sink and the residual energy, reducing the chances of void creation, which in turn results in increased packet delivery ratio and low end-to-end delay. The drawbacks of this scheme include the requirement for location estimates, which adds overhead. Moreover, node mobility is not considered. 

##### CSQSR

Reference [40] presents a cross-layer quality of service routing approach. In order to overcome the QoS determination problems due to non-cross layer framework for routing, the authors propose a cross-layer approach that enables cross-layer communication between physical, datalink, and network layers. This architecture enables the identification of links that meet certain QoS requirements. These links are used by the network layer routing protocols to establish efficient routing paths that ensure better energy efficiency and low end-to-end delays. A routing protocol termed Wireless Acoustic Line Link or WALL is proposed that establishes end-to-end routing paths based on link level information received from lower layers. The paths are established based on the end-to-end QoS requirements. The proposed protocol shows good scalability in terms of network lifetime, as overall residual energy increases with increase in the number of nodes. However, scalability suffers in the case of network throughput, which reduces with increase in the number of nodes.

##### PCR

PCR [41] proposes a joint design of opportunistic routing and power control aimed at achieving better end-to-end packet delivery ratios. The protocol operation can be divided into three main parts, the first of which is Neighbor discovery, during which nodes transmit beacons using every available transmit power level. This phase aims at populating tables of neighborhood based on the transmission power required to reach a neighbor. The neighbor discovery phase is followed by the candidate relay nodes selection phase. This phase selects a set of neighboring sensor nodes that can forward data to the destination node, based on every transmit power level, and the resultant energy consumption. Inclusion of energy consumption helps in cutting out high power transmissions. The third part coordinates the transmission of selected forwarders to achieve fair energy dissipation among the forwarders. In PCR, multiple beacon transmission (using all available power levels) increases communication overhead, contention, and energy consumption. Moreover, if mobility is considered, then the neighbor discovery phase should be carried out periodically. Thus, periodic transmission of beacon with all possible transmission power levels will incur even higher communication overhead, which will result in higher contention and higher energy consumption.

##### EGBLOAD

Reference [42] proposed three routing schemes: (1) corona-based energy grade (EG) & balance load distribution or EGBLOAD, (2) EG w/o corona, and (3) depth adjustment (DA) w/o corona. These schemes collaborate to achieve high energy efficiency and prolong network lifetime by intelligently distributing traffic load among the nodes. The node battery is initially divided into energy grades, based on range measure between the sender and the destination. EGBLOAD adaptively regulates the transmission power according to the energy level of the forwarder, traffic load on the forwarder, and distance of the sender from the forwarder. Energy grade (EG) w/o corona helps in even distribution of traffic load, and in reducing congestion at forwarders by dividing data packets into three sizes i.e., large, medium, and small. Depth adjustment (DA) w/o corona aims to avoid voids by adjusting the depth of the forwarder, without affecting its connection with existing neighbors. Moreover, the proposed scheme achieves balanced load distribution by considering residual energy while choosing forwarders.

##### BEAR

BEAR proposed in [43] aims to prolong network lifetime. This works in 3 phases: (1) Initialization phase: in this phase, nodes share their location and information related to their levels of residual energy; (2) Tree building phase: In this phase, the location information obtained in the initialization phase is used to choose a) neighbor nodes, and b) facilitating and successor sensor nodes, depending on a cost function value. To balance the energy consumption between facilitator and successor nodes, the proposed protocol chooses those nodes whose levels of residual energy are higher, compared to the network’s average residual energy level. (3) Selection of a relay and transmission of data packet to that node. BEAR improves overall load distribution by dividing the network into sectors, and achieving intra-sector and inter-sector energy balancing. However, it has certain drawbacks, such as the 4-way handshake in the neighbor-finding phase, which increases overhead. Moreover if a hello message does not receive a response, the message is retransmitted with increased transmission range, which increases contention and communication overhead even further.

##### SiM-RPO & CoSiM-RPO

**Figure 10:**
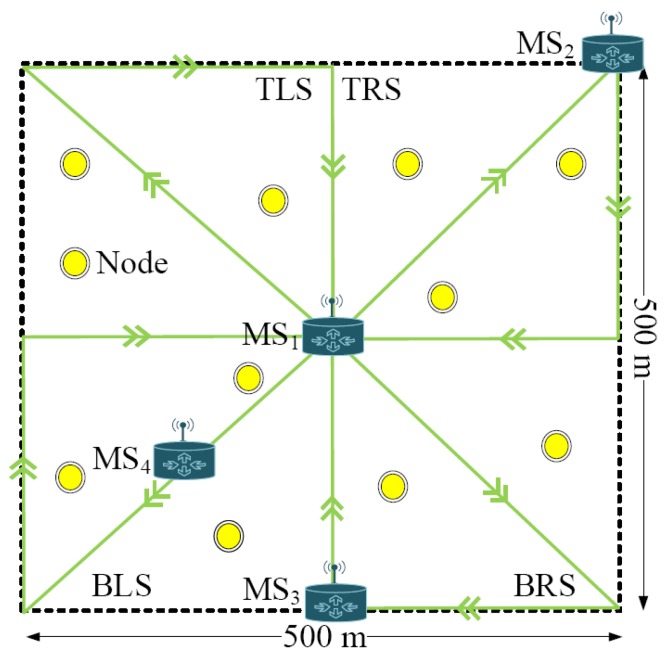
Proposed Network Model [44].

Reference [44] proposes two routing schemes, SiM-RPO and CoSiM-RPO, in order to improve energy efficiency by employing single-hop and two-hop data forwarding. The network area is divided into four equal parts. Each part has its own mobile sink, which traverses its assigned area on a triangular traversal path (Figure 10). 

Nodes transmit their stored data to a mobile sink upon contact through one-hop communication. However, the authors argue that due to the harsh underwater environment, the data may be received in error. To cope with this problem, CoSiM-RPO is proposed. In CoSiM-RPO, the receiving mobile sink checks the received data for errors. If the errors are above a certain threshold value, the mobile sink requests transmission of the same data from other nodes (relay nodes) that might have received the data when it was being transmitted by the sender to the sink. The relay node transmits the data to the sink node. Based on the newly received packet, the sink measures the error. If a certain threshold is satisfied, the transmission is considered successful; otherwise, the packets are dropped. CoSiM-RPO improves the chances of successful delivery. However, if a certain node does not have any other neighbor, the technique may not be effective. Moreover, the number of neighbors also has impact on the bit error rate of the received packet. Data is collected by mobile sinks directly from the sensor nodes. This eliminates the disadvantages of multi-hopping, such as voids and link level reliability issues, neighbor discovery overhead, etc. However, the nodes hold data until a mobile sink is in range. This makes the proposed protocol unsuitable for real-time applications, such as surveillance and target detection, if an ample number of mobile sinks are not employed. Moreover, the path determination for mobile sinks in the case of the mobile network becomes more complex.

### 3.2. Localization-Free Routing Schemes 

Localization-free routing schemes do not require the position information of nodes in order to determine routes for data forwarding. Table 2 analyzes these protocols based on a number of parameters explained in Section 1. We can categorize localization-free routing schemes based on whether or not they consider node mobility.

#### 3.2.1. Localization-Free Routing Schemes with Mobility Consideration

This section surveys the localization-free routing schemes that consider the limited or free mobility of sensor nodes, relay nodes, and/or sink nodes. 

##### EVA-DBR

**Figure 11:**
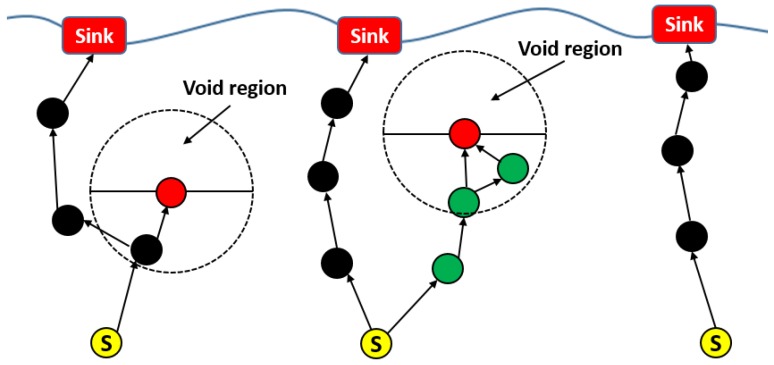
Void regions and trapped nodes problem.

Energy efficient void avoidance depth-based routing (EVA-DBR) [46] is a stateless routing technique. The protocol works in two phases. In the updating phase, nodes transmit information, such as depth of node, node type, etc. to their neighbors, which use the received information to update their neighborhood tables. Base on the neighborhood tables, forwarding decision are made during the routing phase. Void regions and trapped nodes (Figure 11) are proactively detected and avoided while making forwarding decisions. The connectivity holes and trapped nodes are identified. The process is carried out locally, and does not involve extensive messaging, thus resulting in reduced communication overhead, and better scalability. Moreover, EVA-DBR improves its reliability in sparse area by adjusting its forwarding area based on how dense is the area ahead. This also results in better energy efficiency by throttling duplicate packets in denser parts of the network. However, it does not take residual energy into account while making forwarding decisions, which may result in disbalanced energy dissipation.

##### EECOR

EECOR [47] is a cooperative opportunistic routing technique that aims to achieve improved energy consumption. When a node has a packet to send, it selects a set of relay nodes based on their depth. This is followed by applying a fuzzy logic-based relay (FLRS) selection scheme on the selected set to choose the best relay nodes for each hop from source to the sink node. The packet is forwarded to the best selected relay node. This process is carried out at every hop, until the packet is finally delivered to the sink node. Holding time (Equation (16)) is used in order to avoid collision between transmissions of neighboring nodes. The holding time is determined based on two factors: (1) a neighbor’s fitness factor, which is calculated based on depth, and (2) the propagation time between the current sender and the candidate relay nodes in its neighborhood. EECOR achieves high packet delivery ratios and energy efficiency. However, delay may be added due to the forwarder selection process, which involves repeated inspection and selection of the forwarder set:

(16) $$TH_{r}{}_{j}=\left(1-\mu_{r}{}_{j}\right)\left(\tau_{max}\right)+\frac{R_{\max-}\left|\underset{d_{ir_{j}}}{\to }\right|}{C}$$

where C, $R_{max}$, and $\tau_{max}$ are the speed of sound in water, max transmission range and max propagation delay, respectively. $\underset{d_{ir_{j}}}{\to }$ is the distance between the source node and its neighbor relay node.

##### MMS

MMS [48] is a multi-sink routing protocol. It works in two phases: a layering phase, and a communication phase. During the layering phase, concentric layers are formed around each sink, which are refreshed periodically. The layered structure is refreshed based on the packet delivery rate. If the packet delivery rate falls below a certain threshold, the sink node refreshes its layered structure. Sensor nodes may be situated in the layered structure of one or more mobile sink nodes. The communication phase deals with the selection of the sink node to which a node should transfer its data, and formation of the path towards that sink node. The path is formed by selecting the relay node based on a criterion that aims to reduce energy consumption while improving end-to-end delivery ratio. The drawback of this scheme is that it does not address the connectivity void problem. Secondly, it takes into account only depth/hop-count as next hop selection criteria. This results in improving end-to-end delay. However, the criteria lack parameters such as link condition, which ensures reliability, and residual energy, which prevents connectivity voids by guaranteeing balanced energy dissipation. 

##### SUN

SUN [45] is a reactive source routing scheme. Its reactive nature makes it a less control messaging intensive protocol, which enables it to save the scarce underwater acoustic bandwidth. Moreover, by employing source routing, SUN allows the source node to decide the whole route from source to destination, thereby eliminating delays and control messaging incurred due to hop-by-hop relay selection. Furthermore, because of its dynamic design, it can adjust to significant topology changes caused by the mobility of nodes, variations in the channel conditions etc. SUN employs a cross-layer mechanism. It buffers the packet received from a lower layer of the protocol stack, as well as the data to be transmitted. In addition, it implements another cross-layer feature in the form of a Stop-and-Wait ARQ mechanism that enables it to detect unpredictable routes and overloaded links. SUN protocol reduces energy consumption and communication overhead. However, it experiences high end-to-end delay, due to path discovery and maintenance activity. 

##### EAVARP

EAVARP [49] is an energy-aware and void-avoidable routing protocol designed for 3D mobile networks, which works in three phases: Initialization phase, layering phase, and data collection phase. The initialization phase sets the IDs, energy levels, layer information, and initial routing table of the sink and ordinary sensor nodes.

The layering phase involves forming a concentric structure around the sink node. The phase is initiated by the sink node, which broadcasts a layering packet that is received and rebroadcasted by node. This phase determines the distances of sensor nodes from the sink node in terms of hop counts. Neighborhood information of sensor nodes based on the residual energy levels of the neighboring nodes is also collected. In the data collection phase data is forwarded using an opportunistic data forwarding scheme (ODFS). Moreover, the transmission capacity of neighboring nodes (determined during the layering phase) is also taken into account while making forwarding decisions. By using residual energy as part of the forwarder selection criteria, EAVARP achieves load balancing, which results in preventing connectivity void creation and prolonging network lifetime. However, it achieves low packet delivery ratio for a smaller number of nodes. 

##### Co-EEORS

Co-EEORS [50] uses cooperative routing to find energy efficient routes from sensor nodes to sink node. Forwarding nodes or relays are selected based on two parameters: depth, and location value. Depth is measured using pressure sensors, whereas the location value (which does not refer to the geographic location of a sensor node) is measured in terms of a node’s distance from the surface sink node. The sink node transmits beacons periodically, in order to help sensor nodes update their location estimates. Based on the two values, the node closest to the sink node is selected as a relay node. Once a relay node is selected, data is forwarded to it, which relays it to the destination. The destination node sends acknowledgment to the source node in the case of a successful reception, or requests retransmission in the case of a failure. CoEEORS adds reliability by incorporating acknowledgements for received packet in the protocol design. Acknowledgement informs the sender of successful reception, or requests retransmission in the case of erroneous reception. However, acknowledgements add additional overhead. 

##### SORP

**Figure 12:**
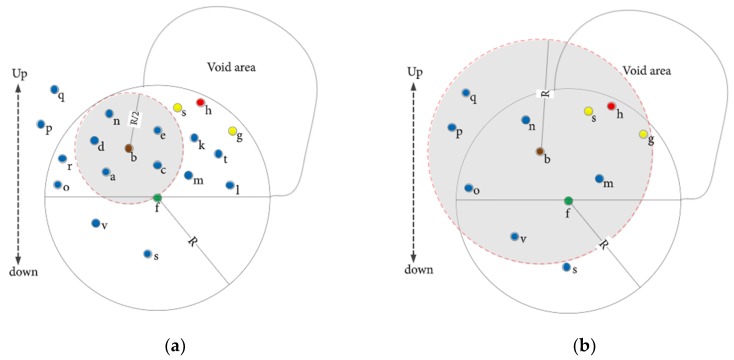
(**a**) Forwarding area in a high density network, (**b**) Forwarding area in a low density network [51].

SORP [51] is a depth-based stateless opportunistic routing protocol. This focuses on the issue of voids and trapped nodes faced by routing protocols in UWSNs. It uses information received from neighboring nodes to detect communication voids. It can also detect trapped nodes locally, and exclude them in the route establishment process, to ensure high end-to-end delivery ratio. The protocol works in two phases: updating, and routing phases. The updating phase is carried out periodically, in which information is shared between neighbors. The routing phase deals with routing data to the sink node while selecting the best forwarders, based on the information collected during the updating phase. The information is used to detect and exclude voids and trapped nodes from the route establishment process, using a passive participation approach. SORP proposes a technique to employ an adaptive forwarding area (Figure 12). The forwarding area is adaptive in the sense that it can be changed, and/or its size can be altered, based upon the density of nodes in the local area and position of the candidate relays, thereby improving the reliability and energy efficiency of the network. The impact of variable delays and shadow zones on the performance of the proposed technique has also been analyzed by the authors. SORP achieves a good packet delivery ratio (PDR) in dense networks; however, PDR can be very low in the case of sparse networks. The same is true in the case of end-to-end delay. 

##### RMCN

Reference [52] proposes RMCN, which is a radius-based courier node routing protocol for long-term underwater sensing applications. RMCN divides the network into two types of areas: sink areas, which constitutes one-quarter of the total area, and node areas, which constitute the remaining three-quarters of the total area. Sensor nodes in the node area are equally divided into static and mobile courier nodes. Forwarding decisions are based on four metrics, which are: depth of nodes, energy levels of nodes, track ID, and a cost function. The packet delivery ratio is improved through efficient coordination between courier and static nodes. RMCN improves energy efficiency by avoiding data-flooding and the creation of multiple copies of the forwarded packet. However, the drawback of RMCN is that it assumes that nodes are deployed according to a set criterion. Therefore, random deployment of nodes, (which may be the case in many applications), may result in reduced performance of the protocol.

##### RECRP

RECRP [53] is a single copy, cross-layer routing protocol. The aim of this protocol is to enhance 2-hop delivery, while achieving better energy consumption. To this end, the protocol proposes a minmax technique that can adjust power and frequency dynamically according to the requirement. The minmax technique is used in conjunction with a mixed integer linear programming model to design energy efficient forwarding. Moreover to improve routing, the protocol is designed as a cross-layer framework. This enables information to be acquired, like Doppler shift from lower layers for estimating relative speed among underwater sensor nodes, and regulating transmission power and frequency to achieve better transmission efficiency. The protocol works in two main phases: Routing table update phase, and routing phase. The routing table update phase is carried out periodically. Sink nodes initiate the update process by broadcasting a routing update phase. This phase is responsible for updating the routing table information of nodes. In the routing phase, transmission power, frequency, and forwarding nodes are selected based on the routing tables, to achieve energy efficient forwarding. RECRP achieves moderate end-to-end delay and packet delivery ratio for a small number of nodes. However, the two parameters improve as the number of nodes increases. A drawback of this protocol is the excessive energy spent during the periodic routing update phase. During this phase, all underwater sensor nodes broadcast with the maximum power, which not only increases energy consumption, but also expands the contention domain, thus increasing delay and incurring further energy consumption. Periodic transmissions with maximum power may quickly drain the network energy.

##### LF-IEHM

**Figure 13:**
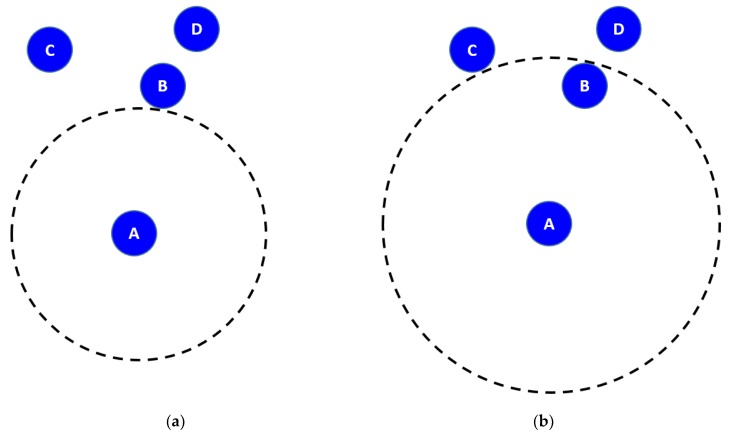
Adaptive neighbor determination. (**a**) No neighbor in the range of node A, (**b**) Node A increases power to be able to access neighbors.

LF-IEHM [54] is a localization-free depth-based routing protocol. In LF-IEHM, forwarding decisions are made based on depth of nodes. The “in range” nodes nearest to the water surface are chosen as candidate relays. If two or more candidate relays have the same depth, the relay node with the smallest response time (which is mainly dependent on the distance from the source node) is chosen as relay. LF-IEHM improves packet delivery ratio and energy efficiency in the routing process using two strategies. Firstly, it alters its transmission power in order to avoid voids. If a node is stuck in a void, it increases its transmission power to reach out to a sink node, or to a node with smaller depth that can act as relay node (Figure 13). This technique is intended to achieve small end-to-end delay, by finding out the shortest path to sink nodes on the water surface. Secondly, in order to reduce interference among transmitting nodes, every transmitting node is assigned a unique holding time given by Equation (17):

(17) $$TH=\frac{N_{j}\left(PrL_{i}-PrL_{j}\right)}{C\left(\frac{E_{initial}}{E_{Current}}\right)}$$

where $N_{j}$, $PrL_{i},\;PrL_{j}$, $E_{initial},\;E_{Current}$ and *C* denote the no. of neighbors, the pressure level of the sender, the pressure level forwarder, initial energy level of the forwarder, current energy level of the forwarder and the speed of sound in water, respectively.

This strategy of assigning unique holding time to every transmitting node, minimizes interference, which results in increased successful delivery of packets, thus improving packet delivery ratio and energy efficiency. Moreover, this strategy also improves end-to-end delay by improving hop-to-hop successful delivery probability. The scheme improves packet delivery ratio in sparse networks using a “variable transmission power” strategy; however, this may increase power consumption as the network becomes sparser. Furthermore LF-IEHM considers only depth as the forwarder selection criterion. This (especially in the absence of considering residual energy) will result in early death of the nodes near the surface, thus creating connectivity voids.

##### EDBF

EDBF [55] is a depth-based forwarding mechanism. It selects next hop based on three parameters: (1) Depth of the forwarding node: a node whose depth is smaller than the sender is selected as a forwarder; (2) Residual energy: this prolongs network lifetime by achieving balanced load distribution, which results in preventing the creation of communication void; and (3) Forwarding quality; the forwarding quality of a node is determined based on link or node failures, the presence or absence of connectivity voids, etc. Inclusion of this metric improves the successful forwarding of data packets. EBDF reduces communication overhead; however, due to its simple design, especially in determining forwarding quality, the end-to-end delivery ratio does not improve significantly. 

##### RE-PBR

RE-PBR [56] is a depth-based routing protocol. This scheme proposes the use of a triangle metric to determine the quality of links. The triangle metric determines link quality not only based on successfully received packets, but also takes into account the packets that are not received successfully. An information dissemination scheme is proposed to share information among neighboring nodes. This information is used to select a forwarder during the data transmission phase. Data forwarding decisions are made based on three parameters, which are: (1) Link quality between the sender and the candidate forwarder. This parameter ensures the reliable delivery of packets. (2) Residual energy of the candidate forwarder, which ensures load balancing; and (3) Depth, which improves end-to-end delay (Equation (18)). RE-PBR uses an implicit Ack mechanism in order to reduce communication overhead and improve reliability. Implicit Ack refers to a mechanism in which the receiving nodes do not send ACKs explicitly. Instead, the sender node assumes successful transmission to the next hop node if it overhears the same packet being retransmitted by another node. Moreover, the use of multi metric data forwarding by RE-PBR improves its overall performance by preventing void creation, balancing energy dissipation, achieving reliable communication and end-to-end delay. A drawback of the proposed scheme is that it is designed for grid networks, and in the case of random network topologies, may result in performance degradation. Moreover, for link quality estimation, it requires transmission of packets other than the usual hello packet, thus increasing communication overhead:

(18) $$PathCost\left(x,y\right)=\left(1-\frac{RE_{y}}{TE}\right)+\left(1-\frac{∆d_{\left(x,y\right)}}{∆d_{max}}\right)$$

where $RE_{y}$ and TE denote the residual energy and total energy of node y respectively. $∆d_{\left(x,y\right)}$ denotes the link quality between sender *x* and forwarder *y*. $∆d_{max}$ is a system parameter that is set based on the environment.

##### DQELR

**Figure 14:**
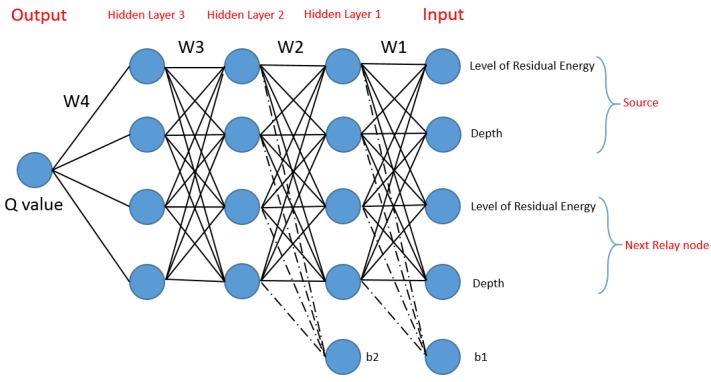
Multi-layer perception (MLP) neural network model.

DQELR [57] is a latency- and energy-aware routing scheme that aims to prolong network lifespan. It implements a deep Q network (DQN) algorithm using both on-policy (i.e., training the neural network online), and off-policy (i.e., training the neural network offline) techniques to route adaptively according to network conditions (Figure 14). In order to reduce overhead, the authors use a hybrid approach, where unicast and broadcast communication are combined. With stringent latency constraint and less energy consumption, the lifespan of the network is prolonged by picking forwarders having the highest Q value. Moreover, DQELR can handle changes in topology by implementing an on-policy technique to find new routes upon disruption of the current route. DQELR achieves improved network lifespan with low latency and low energy consumption.

##### TBRS

TBRS or the time-variant load balancing scheme proposed in [58] aims to extend network lifetime by designing a balanced distribution of network traffic among nodes. Sensor nodes are deployed based on a distinct deployment scheme. The deployed nodes are anchored with the help of long ropes, and can be pushed to the water surface with the help of buoys. Each sensor node can alter its Tx power up to three different levels, thus achieving different ranges. This flexibility enables a transmitting sensor node to reach a certain next hop node having related weight and Tx power, resulting in fair energy depletion, and consequently mitigating the energy sinkhole issue. To achieve this, the authors have developed a comprehensive analytical model. Optimal weights that define traffic load on each candidate next hop node are derived taking into account the mobility pattern of sensor nodes. The node with the most optimal weight is selected for transmission. Simulation results show that data traffic can be fairly distributed among nodes, so long as the nodes can switch between three different transmission power levels. Moreover, the proposed scheme achieves an extended network lifetime and improved energy efficiency, compared to the nominal range-based data forwarding.

##### RAR & RACAA

**Figure 15:**
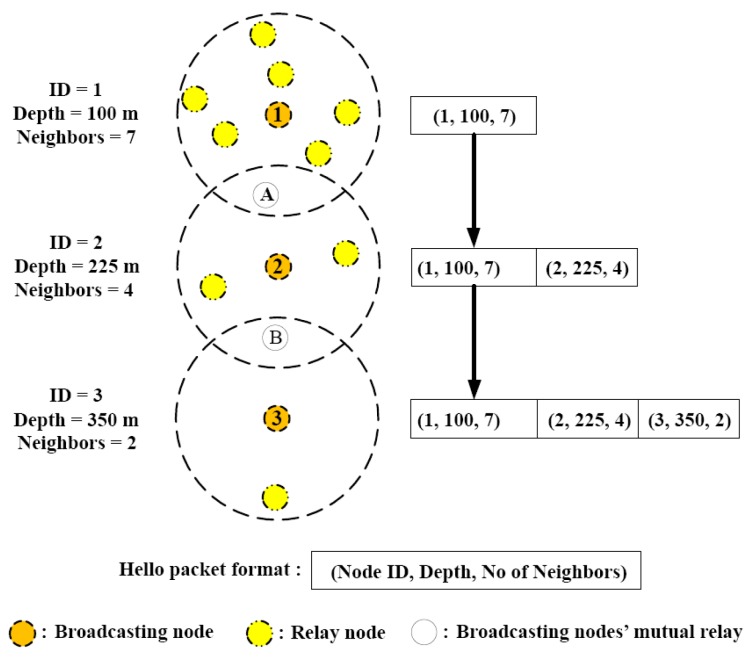
Information sharing strategy between one and multi-hop neighbors.

Reference [59] proposes two routing schemes, namely the reliability aware routing scheme or RAR for applications that require reliability and low end-to-end delays, and the reliability aware cooperative routing with adaptive amplification or RACAA, for applications that require reliability, but are delay tolerant. RAR involves the establishment of reliable end-to-end connected routes from source to destination based on neighborhood information (Figure 15), before the actual transmission of data packets. Multiple routes are considered, and the route with the highest probability of successful packet delivery is selected for transmission. However, the quality of the underwater channel is prone to fluctuation, and the performance of the pre-determined routes of RAR may fluctuate too. Therefore, RACAA is introduced to compliment RAR. In RACAA, a forwarder may increase its transmission power if the error in the data that it received for forwarding is higher than a certain threshold value. The rest of the operations of RAR and RACAA are the same. Neither of the proposed protocols require location information, which is a challenging task in the underwater environment, and involves further communication overhead. This makes the design of RAR and RACAA simpler, compared to other routing protocols that do require location information in order to route data packets. Moreover, adaptive amplification based on error probability (which is based on channel conditions) improves reliability and the end-to-end delivery ratio. Reliability is further improved by combining copies of the same packet (at the destination node) from the source and a relay node. 

##### EP-VIR-3 & BF-SPR-3

**Figure 16:**
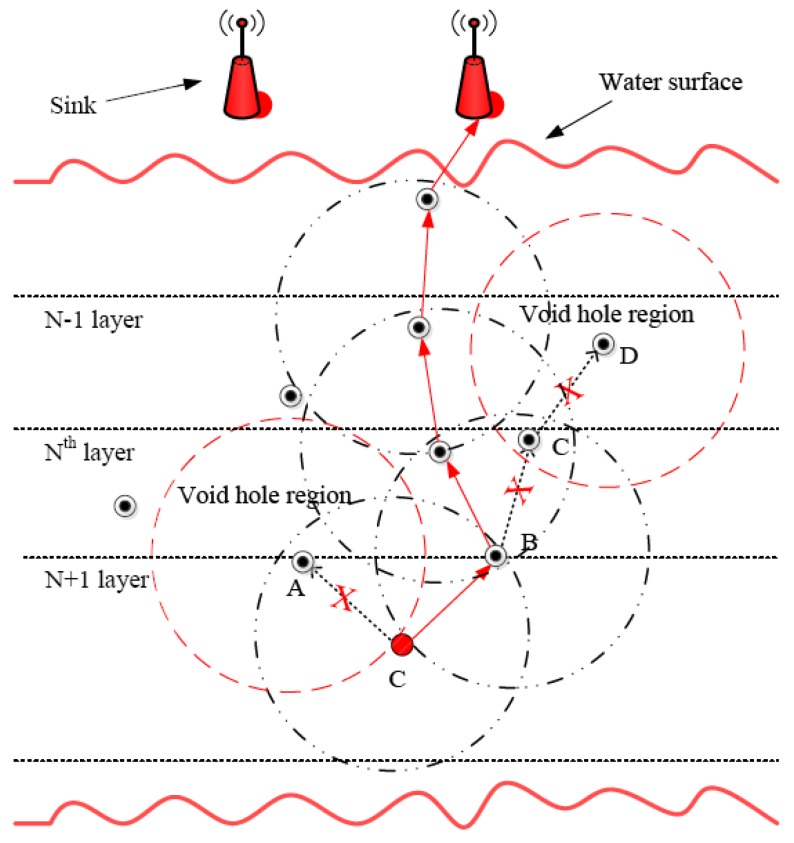
Selection of relay node in EP VIR 3 and BF SPR 3.

Reference [60] proposes the energy-efficient path-based void hole and interference-free routing (EP-VIR-Three) and Bellman-Ford shortest path-based routing (BF-SPR-Three) routing protocols. These two proactive routing protocols adopt a sender-centric approach to improve reliability and reduce the likelihood of void occurrence. In order to improve the packet delivery ratio and avoid the void problem, a sender node chooses a subsequent relay node based on info about its three-hop neighbors. The info is based on difference in the depths of the current and the preceding node. In the case of BF-SPR-Three, the subsequent relay node is chosen using the Bellman Ford technique, which finds the shortest and fastest routes for routing data. Moreover, the binary tree method through horizontal layering is used to ensure reliable delivery of data. Additionally, this technique also resolves the routing loop problem. It prevents voids by taking into account the availability of 3-hop neighborhood of the forwarder (Figure 16). Moreover, energy consumption is reduced by selecting the node that is the least hops away from the sink node. The selection criteria for forwarding node also includes the number of neighbors of the candidate forwarder. The smaller number of neighbors of the candidate forwarder increases its weight in the cost function, thus minimizing energy consumption by minimizing participating nodes.

#### 3.2.2. Localization-free Routing Schemes without mobility consideration

This section surveys the localization-free routing schemes proposed for static underwater sensor networks.

##### EnOR

Energy-aware opportunistic routing (EnOR) [61] aims to prolong the lifetime of the network by distributing the traffic load fairly across the network, thus avoiding node failure due to energy drainage. The protocol carries out routing in two steps. In step 1, a set of potential forwarders is determined based on a fitness value (Equation (19)), which is a function of the depth of the candidate forwarder relative to the sender, its residual energy level, and quality of the link between the sender and the candidate forwarder. In step 2, the sender transmits the packet along with the list of candidate forwarders. The list is sorted according to priority determined through the fitness value. The node with the highest priority is assumed to be the next forwarder. In order to regulate the transmission by candidate nodes, EnOR defines a parameter called “packet holding time” (Equation (20)), i.e., the time for which a candidate node will hold a packet before forwarding. Packet holding time is a function of the priority of the candidate node and the distance between the sender and the candidate node. This ensures that redundant transmissions by low priority nodes are avoided. EnOR achieves load balancing and increases the percentage of alive nodes by including the residual energy of nodes in the selection criteria for forwarders, thus ensuring that nodes at shallower depth levels do not rapidly run out of energy. However, communication overhead, incurred to inform neighbors about current energy levels and depths, may be a concern in the case of dense networks:

(19) $$F_{j}=P_{j}\times p\left(d_{j},m\right)\times \frac{RE_{j}}{RE_{initial}}$$

(20) $$TH\left(p\right)=\{\begin{array}{c} \frac{CR-d_{max}}{C}\;,\;\;if\;p=1\\ \frac{CR+p\times d_{max}}{C},\;if\;p>1 \end{array}$$

where in Equation (19) $P_{j}$ is the probability of packet advancement by neighbor *j*, $p\left(d_{j},m\right)$ is the probability that a packet transmitted by node *i* will be delivered node *j*. $RE_{j}$ and $RE_{initial}$ represent remaining and initial levels of energy of node *j* respectively. 

In Equation (20), *CR*, *d_max_*, p and C denote communication range, max distance between the sending node and candidate forwarders, priority of a candidate forwarder based on its position in the packet header and speed of sound in water respectively.

##### QA DFR AA & QA DFR TA

**Figure 17:**
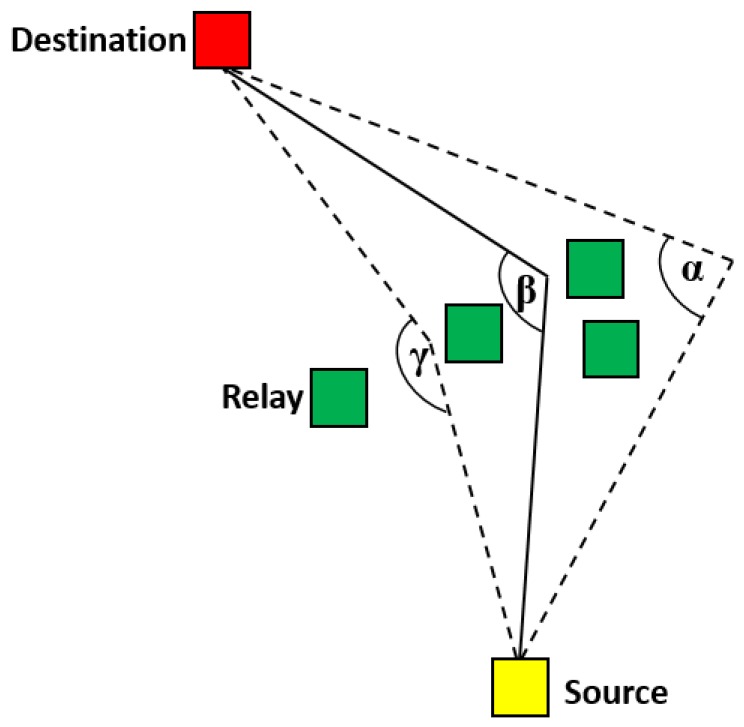
Base angle adjustment to alter flood area.

Reference [62] proposes two enhanced versions of the DFR [63] protocol, namely QA DFR AA i.e., QoS-aware DFR with angle adaption, and QA DFR TA i.e., QoS-aware DFR with threshold adaption. These protocols are aimed at meeting the QoS requirement in a more dynamic fashion. In both the techniques, the sink node measures the end-to-end packet delivery ratios, and updates the sensor nodes through a hello message. The receiving sensor nodes compare the received value with the QoS requirement. If the end-to-end packet delivery ratio is less or more than what is dictated by the QoS requirement, the sensor node adjusts its flooding area, based on the gap between the two values. QA DFR AA adjusts the flooding area by adjusting its base angle (Figure 17). The new base angle is the function of the previous base angle and an adjustment value. The adjustment value in turn is a function of the QoS requirement and the packet delivery ration of each path. In contrast to QA DFR AA, QA DFR TA updates a threshold value, instead of the base angle. Moreover, QA DFR TA adjusts the flooding area locally, unlike QA DFR AA, which adjusts the flooding area all the way from source to destination. Besides the above mentioned two techniques, the authors also proposed a holding time technique for current DFR. Packet holding time is subjected to link quality. Nodes with better quality link have short holding time, as compared to longer holding time in the case of nodes with bad link quality. This results in reduction in overhead. The drawback of this protocol is that it does not take into account mobility and connectivity voids, which result in decrease of packet delivery ratio, and increase in the end-to-end delay. 

##### JARDCM

**Figure 18:**
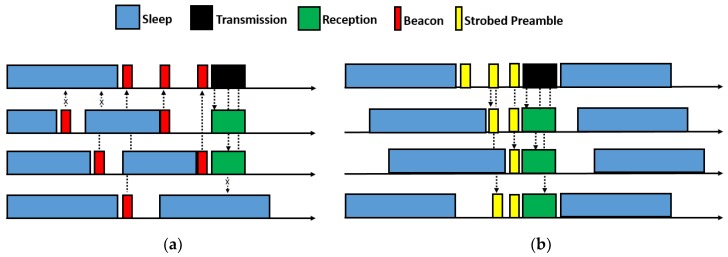
(**a**) LPP duty cycling, (**b**) Strobed LPL duty cycling.

Reference [64] proposes a symbiotic framework of duty cycling and any path routing to address the challenges of high energy requirement and resultant short life span of underwater sensor networks. The model is aimed at improving the end-to-end packet delivery ratio (PDR) and energy efficiency, while reducing end-to-end delay. To achieve the objective, the authors propose two novel techniques to augment asynchronous duty cycling protocols: LPL (or low power listening), and LPP (low power probing) (Figure 18), which enables sensor nodes to listen and send probes with less power, and thus save energy. Moreover, an analytical frame work is presented that can evaluate performance of the symbiotic designs, such as proposed in this work. The two duty cycling methods proposed in this paper i.e., LPL and LPP, help in improving the network lifetime by conserving energy. Moreover, the symbiotic framework of LPL and any path routing is shown to be most suitable for short-term real time applications, as this design achieves acceptable packet delivery ratio, while reducing energy consumption. 

##### HYDRO

HYDRO [65] is an energy harvesting aware routing protocol. It makes forwarding decisions and relay node selection based on four criteria, which are: the current energy level of a node, predicted amount of energy to be obtained through energy harvesting, channel conditions, and macro level energy available along the whole path from source to destination. The protocol smartly combines knowledge about available energy resource and the amount of energy that can be harvested in the near future, to make more flexible routing decisions, thus improving the packet delivery ratio and energy consumption. A multi model approach is proposed for energy harvesting that enables the shallower nodes to harvest solar energy, whereas deeper nodes capitalize on underwater currents to harvest energy. HyDRO achieves a high level of fairness by delivering data from all nodes. However, it suggests that nodes that are low on charge will go to all-off stage, and when they have harvested enough energy, turn on again. This will create voids when a node is in all-off stage. The authors do not explain how these voids will be handled.

##### DVOR

DVOR [66] aims to solve two major problems of opportunistic routing in underwater sensor networks, which are connectivity voids, and longer alternate path selection for routing. The problems are addressed by introducing distance vector based opportunistic routing. The distance vectors, initially estimated using short query messages, find the minimum number of hops from a source sensor node to a sink node. Then opportunistic routing is applied based on the estimated distance vectors, to route packets from source node to sink in a hop-by-hop fashion. This technique provides two advantages: Firstly, the distance vector can identify void area, thus these areas are avoided while making forwarding decisions. Secondly, due to shortest path routing, the problem of relatively longer detour is also eliminated. DVOR improves performance, as it does not require information about neighboring nodes while making forwarding decisions, thus reducing communication overhead. Moreover, it does not require carrying a list of candidate forwarders in the packet header. Different from the existing OR protocols, DVOR does not need any information about its adjacent nodes, and does not need to carry a relay candidate list in the packet header, thus reducing signaling overhead. Avoidance of long detour improves end-to-end delay, whereas void avoidance improves packet delivery ratio. However, DVOR ignores link quality and residual energy, which result in a lack of reliability and unbalanced load distribution, respectively. 

##### DMR & CoDMR

**Figure 19:**
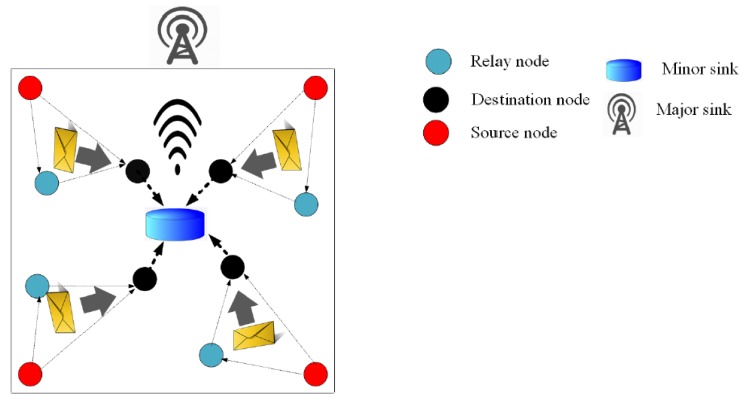
Cooperative forwarding.

Reference [67] proposes two routing protocols, delay minimization routing or DMR, and cooperative DMR or CoDMR. The aim of the two protocols is to reduce the delay induced due to long detour of packets in multi-hop routing protocols. To achieve this, DMR partitions the network into four equal parts. A sink node is deployed at the center of each part All of these sensor nodes in each part that can access their sink node directly send their packets to the sink node through one-hop forwarding. However, farther nodes send packets to the sink node through multi-hop forwarding. The authors argue that dividing the network into four parts and deploying a sink in each part will reduce latency, as compared to the case when data has to be routed through multiple hops to the sink nodes on the ocean surface. DMR can face reliability issues due to transmission of data over a single link. To cope with this, CoDMR is proposed. In CoDMR, the senor nodes that can access the sink nodes directly send their packets directly to the sink node. However, if the sink node is out of communication range of the sender, packets are sent through cooperation with a single relay (Figure 19). The node having lowest distance with respect to the sink node is considered as the destination node. The proposed schemes solve the long detour to surface sinks and the resultant latency, to some extent. However, the long detour issue due to multi-hop forwarding, which may occur inside partitioned parts of the network, is not addressed. CoDMR adds limited improvement in reliability by merging two copies of the same packet. However, the next hop selection criterion considers only distance from the sink node. The channel condition is ignored, which may create reliability issues, despite the merging of two copies of the same packet to improve reliability. 

##### GEDPAR & E2EVHR

**Figure 20:**
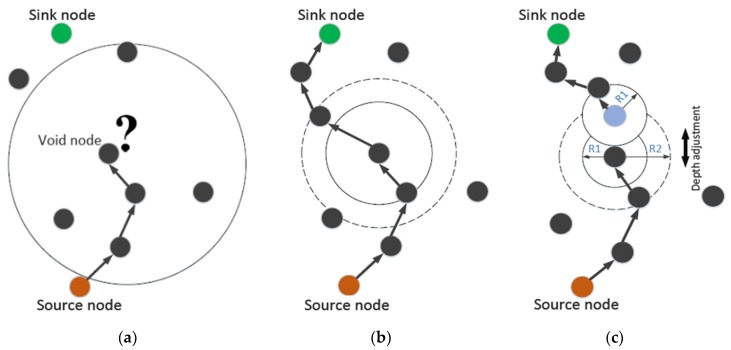
(**a**) void area, (**b**) Adjustment of transmission Power, (**c**) Adjustment of depth.

Reference [68] proposes two routing techniques, GEDPAR and E2EVHR. GEDPAR is basically a combination of GEDAR [69] and LMPC [70]. GEDPAR takes its layering scheme from LMPC. The nodes are deployed in layers of unequal interlayer distance. The size of a layer is inversely proportional to noise i.e., the size of the layer shrinks with increase in noise levels, and vice versa. Whenever a void region is encountered by a node, it increases its transmission range. However, if it cannot reach another node of smaller depth, even with the maximum possible transmission power, it resorts to depth adjustment (Figure 20). This scheme efficiently handles voids at the cost of higher energy consumption. E2EVHR improves LMPC by finding a complete void-free route from source to destination, as opposed to hop-by-hop forwarding of LMPC. This is followed by routing multiple copies of a packet along multiple paths of a predefined binary tree structure. This improves end-to-end delivery of E2EVHR. However this scheme has some demerits. As the packet travels on multiple paths in parallel, a lot of redundant transmissions are incurred that will result in high energy consumption. Moreover, the predefined paths may go stale, due to node mobility.

##### CACR

**Figure 21:**
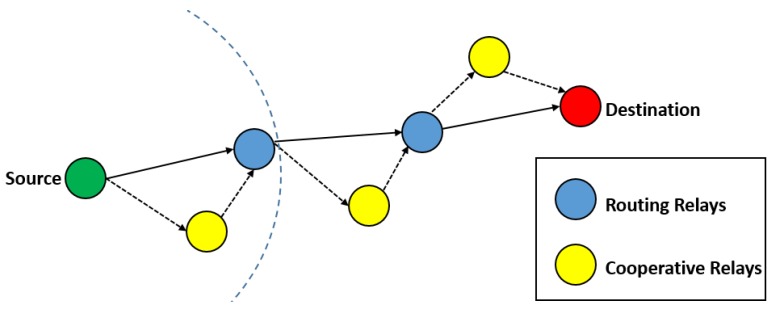
Cooperative routing.

Reference [71] proposes a cross-layer technique for cooperative routing based on channel quality indicators obtained from physical layer. The technique involves simultaneous selection of routing relays, which are responsible for data forwarding, and cooperative relays, which carry out single-hop cooperative communication (Figure 21). Sensors nodes that want to transmit data independently select routing and cooperative relays. The relays are selected from the neighbors of the sender based on the link quality metrics, such as SNR and time of arrival. Moreover, the distance of the sender from the destination in terms of hop counts is also taken into account. Routing relays are selected based on propagation delays and the link’s channel capacity, while the cooperative relay that has least propagation delay from the selected routing relay is chosen to decrease spread delay. As channel conditions play an important role in relay selection, channel asymmetry is taken into consideration to make routing decisions more reliable. The proposed scheme improves reliability even more, by incorporating a cooperative transmission scheme in every hop. However, this reliability comes at the cost of extra transmission, as there is one extra transmission at every hop by the cooperative relay nodes.

##### CEETHCoR

Reference [72] proposes a cross layer, 2-hop routing protocol called the channel aware energy efficient two-hop cooperative routing protocol (CEETHCoR). The design of the protocol includes physical layer, MAC layer, and network layer. In two-hop communication, the source node selects a next-hop node, a relay node, and a 2-hop forwarder. The data from the source is sent to the 2-hop forwarder through a forwarder and a relay for reliability. The protocol operates in 4 phases, which are: (1) network initialization, during which information about neighbors, such as SNR, number of hops from destination, residual energy levels of nodes, etc. are acquired. (2) RTS/CTS message exchange, which reduces contention, and helps nodes to update their tables and decide to offer themselves as relays. (3) Relay Selection: During relay selection, phase relays are selected based on link quality and symmetry in link quality. (4) Data transmission phase: During this phase, actual data is transmitted and acknowledged. CEETHCoR improves reliability by incorporating a cooperative transmission scheme. Moreover, redundant relay transmissions are controlled by using one cooperative relay transmission over two hops.

**Table 2:** Survey of localization-free routing protocols.

Ref #	Main Consideration	Next Hop Selection Criteria	Mobility	Location Required	Clustering	Connectivity Void Handling	Sink	Deployment
EVA-DBR [45]	Detect and bypass the trapped and void nodes in UWSNs.	Depth, distance from the current sender, should not be a void node or trapped node.	Yes	No	No	Yes	Multi-sink	3D
EECOR [46]	Energy Efficiency	Depth, Energy Consumption Ratio (i.e., ratio of the residual and initial energy), Packet delivery probability of the forwarder	Yes	No	No	No	Single-sink	3D
MMS [47]	Energy Efficiency, Packet delivery ratio	Depth/hop count	Yes	No	No	No	Multi-sink	3D
SUN [48]	Improve routing for networks with unreliable links and mobile nodes	Hop count or SNR	Yes	No	No	No	Multi-sink	3D
EAVARP [49]	Balanced load distribution, void avoidance and network lifetime	Transmission capacity (i.e., the node selected as relay should have enough residual energy for transmission, and it should not be a void node)	Yes	No	No	Yes	Multi-sink	3D
Co-EEORS [50]	Reliability, improved Energy efficiency	Depth and location value (location value does not refer to the geographic location of a sensor node, but is measured in terms of a node’s distance from the surface sink node)	yes	No	No	No	Single-sink	3D
SORP [51]	Void Handling	Depth, the node in question should not be a void or trapped node, and it should be located in the forwarding area	Yes	No	No	Yes	Multi-sink	3D
RMCN [52]	Facilitate network operations for longer periods in risky areas	Residual Energy Distance between Sender and candidate forwarder depth	Yes	No	No	No	Multi-sink	3D
RECRP [53]	Reduce and balance Energy consumption	Node Level (min hop count to sink), Distance between the sender and the forwarder, Residual Energy	Yes	No	No	Yes	Multi-sink	3D
LF-IEHM [54]	Void management and interference mitigation	Pressure level (depth) Response time (a function of mainly Distance between sender and the candidate forwarder)	yes	No	No	Yes	Single-sink	3D
EDBF [55]	Load Balancing, Void Avoidance	Residual energy, depth, and historical forwarding conditions	Yes	No	No	Yes	Multi-sink	3D
RE-PBR [56]	End-to-end delivery, Reliability, load balancing	Depth, Residual Energy, Link Quality	Yes	No	No	No	Multi-sink	3D
DQELR [57]	Prolong network lifetime	Q value (which is based on Residual energy, depth)	Yes	No	No	No	Single-sink	3D
TBRS [58]	Energy Sink hole problem, load balancing, prolong network lifetime	NA	Yes	No	No	Yes	Single-sink	2D
RAR & RACAA [59]	Reliable end-to-end routing	Predetermined paths selected based on highest probability of success, which is a function of path connectivity and channel conditions	Yes	No	No	No	Single-sink	3D
EP-VIR-3 & BF-SPR-3 [60]	Energy efficiency, interference-free transmission, void hole avoidance, and high Packet Delivery Ratio	Distance from the sender, hop count from sink, minimum no. of neighbors of forwarder node	Yes	No	No	Yes	Multi-sink	3D
EnOR [61]	Extend the network lifetime	Residual Energy, link reliability, depth	No	No	No	No	Multi-sink	3D
QA-DFR-AA & QA-DFR-TA [62]	QoS aware Routing, avoid packet collision and redundant packet transmission	NA	No	No	No	No	Single-sink	2D
JARDCM [64]	Energy Efficiency, Reliable data delivery	Residual Energy, Depth, Packet advancement, delay	No	No	No	No	Multi-sink	3D
HYDRO [65]	Increased network lifetime by exploiting energy harvesting. Improve energy efficiency, latency and PDR.	Residual energy and foreseeable harvestable energy channel quality and a measure of energy availability through the whole route to the sink	No	No	No	No	Single-sink	3D
DVOR [66]	Solving Void and long detour problems	Hop count to sink	No	No	No	Yes	Multi-sink	3D
DMR & CoDMR [67]	Delay minimization	Distance to the sink node	No	No	No	No	Multi-sink	3D
GEDPAR & E2EVHR [68]	Void elimination and network lifetime	Energy consumption	No	No	No	Yes	Multi-sink	3D
CACR [71]	Reliable data delivery	Link quality and hop count to destination	No	No	No	No	Single-sink	3D
CEETHCoR [72]	Energy Efficiency, Reliability	Link Quality, hop count, Residual Energy	No	No	No	No	Single-sink	3D

## 4. Conclusions

In this work, we present a survey of recently proposed routing protocols for underwater acoustic sensor networks. The protocols are analyzed based on an exclusive set of parameters that cover most of the fundamental aspects of a routing protocol. Moreover, the routing techniques of each of the surveyed protocols are also briefly explained, along with their merits and demerits. The surveyed schemes have been divided into two main categories, which are: (a) Localization-based routing protocols, i.e., the routing protocols that need information about the geographic coordinates of nodes to carry out their operations successfully, and (b) Localization-free routing protocol, which refer to the routing protocols that do not need information about the geographic coordinates of nodes to carry out their operations successfully. The protocols in each of these categories are further divided based on mobility.

## 5. Future Work and Challenges

Most of the routing protocols proposed for underwater acoustic sensor networks consider only static nodes and/or anchored nodes with limited mobility, while ignoring scenarios that feature free and unchecked mobility of freely floating untethered underwater sensor nodes. Untethered sensor nodes move freely with the underwater streams. Consideration of such scenarios gives rise to new challenges and demand for drastic changes in the prevalent architecture assumed in most of the routing schemes proposed for underwater acoustic sensor networks. 

For example with free mobility of sensor nodes, network partitioning becomes inevitable, as nodes drift away from each other over time. Network partitioning, which is different from connectivity voids, refers to the scenarios in which free mobility causes the network to split into two or more unconnected parts, thus creating scenarios where either sink nodes are not accessible at all, or the previously estimated routes are obsolete, due to the change in neighborhood information. Moreover, besides network partitioning, the magnitude of the connectivity void problem is also expected to increase. Besides mobility, connectivity voids may also be created due to environmental changes. This happens due to change in propagation characteristic of acoustic waves in different environmental conditions such as change in temperature and salinity of water. This may result in situations where, nodes that were previously accessible are not accessible anymore thus introducing connectivity voids in the network.

Moreover, the changes in propagation speed may also affect accurate location estimation of a node, which in turn may affect the performance of the location-based routing schemes. Another issue related to the free mobility of nodes is the continuously changing topology of the network. For networks in which nodes move in the same stream, the topology may not change in the short run, due to negligible changes in the relative positions of node, because of movement in the same stream. However, in the medium and long run, the topology may change, resulting in inconsistent network density i.e., some parts of the network may have high density of nodes, whereas other parts may be sparse, with many potential voids. This gives rise to the need for routing protocols that can dynamically alter their routing strategy in different parts of the network based on sparsity. This further gives rise to the need to embed mechanisms in the routing protocols to determine network sparsity.

Free mobility of nodes also dictates drastic changes in the network architecture assumed in most of the proposed schemes. This design seems infeasible, as sensor nodes (which move freely with water currents) quickly move far from the sink nodes, and are unable to communicate with the sink. This results in inability to acquire the sensed data. Propelled sink nodes could be used, which are capable of tracking the underwater network, and adjusting their positions accordingly, by moving with the network in such a way that they are always in communication with a certain number of underwater nodes. 

In most of the routing schemes proposed for underwater sensor networks, the nodes near the surface are more often selected as relays due to their close proximity with the sink nodes. This results in almost inevitable earlier energy drainage and consequently early death of such nodes. The connectivity holes and/or partitions thus created hamper reliability of the network. Deeper nodes may experience higher end to end delays, and low packet delivery ratios thus reducing overall utility of the network. This area still needs to be explored. More effective solution should be proposed to balance energy drainage of nodes in the network. 

In order to optimize performance of the network, cross layer design should be investigated. For instance routing decision based on input from physical and mac layer may improve reliability and packet delivery ratios by taking informed routing decisions keeping in view link conditions and traffic load in different parts of the network. A few studies have been conducted on cross layer approaches for routing in UWASNs. However, a major part of the current literature does not consider cross layer approaches. Future research should explore this avenue to look efficient way for improved network performance.
